# Recent advances in the nucleolar responses to DNA double-strand breaks

**DOI:** 10.1093/nar/gkaa713

**Published:** 2020-08-28

**Authors:** Lea Milling Korsholm, Zita Gál, Blanca Nieto, Oliver Quevedo, Stavroula Boukoura, Casper Carstens Lund, Dorthe Helena Larsen

**Affiliations:** Danish Cancer Society Research Center, Nucleolar Stress and Disease Group, Strandboulevarden 49, 2100 Copenhagen, Denmark; Danish Cancer Society Research Center, Nucleolar Stress and Disease Group, Strandboulevarden 49, 2100 Copenhagen, Denmark; Danish Cancer Society Research Center, Nucleolar Stress and Disease Group, Strandboulevarden 49, 2100 Copenhagen, Denmark; Danish Cancer Society Research Center, Nucleolar Stress and Disease Group, Strandboulevarden 49, 2100 Copenhagen, Denmark; Danish Cancer Society Research Center, Nucleolar Stress and Disease Group, Strandboulevarden 49, 2100 Copenhagen, Denmark; Danish Cancer Society Research Center, Nucleolar Stress and Disease Group, Strandboulevarden 49, 2100 Copenhagen, Denmark; Danish Cancer Society Research Center, Nucleolar Stress and Disease Group, Strandboulevarden 49, 2100 Copenhagen, Denmark

## Abstract

DNA damage poses a serious threat to human health and cells therefore continuously monitor and repair DNA lesions across the genome. Ribosomal DNA is a genomic domain that represents a particular challenge due to repetitive sequences, high transcriptional activity and its localization in the nucleolus, where the accessibility of DNA repair factors is limited. Recent discoveries have significantly extended our understanding of how cells respond to DNA double-strand breaks (DSBs) in the nucleolus, and new kinases and multiple down-stream targets have been identified. Restructuring of the nucleolus can occur as a consequence of DSBs and new data point to an active regulation of this process, challenging previous views. Furthermore, new insights into coordination of cell cycle phases and ribosomal DNA repair argue against existing concepts. In addition, the importance of nucleolar-DNA damage response (n-DDR) mechanisms for maintenance of genome stability and the potential of such factors as anti-cancer targets is becoming apparent. This review will provide a detailed discussion of recent findings and their implications for our understanding of the n-DDR. The n-DDR shares features with the DNA damage response (DDR) elsewhere in the genome but is also emerging as an independent response unique to ribosomal DNA and the nucleolus.

## INTRODUCTION

### Nuclear organization

Most of the genetic material in eukaryotic cells is packed in the nucleus with a spatial arrangement that reflects its biological function (1). This strict organization allows cells to coordinate genome-related functions in faithful ways while misregulations of nuclear organization can cause diseases (2). In human cells, predominant structures include chromatin (3), nuclear bodies (4,5) and sub-nuclear domains such as nucleoli (6).

The nucleolus is the largest nuclear sub-structure, with an organization that is tightly linked to its role in ribosome biogenesis (7,8). It contains the most highly expressed genes in the cell alongside repressed heterochromatic sequences. A high density of nucleic acids and ribosomal proteins creates a unique physical environment distinct from other cellular compartments (7,8). These features affect how all basic cellular functions are conducted in the nucleolus including transcription, replication and DNA repair.

### Functions of the nucleolus

The canonical function of the nucleolus is ribosome biogenesis. The synthesis and processing of ribosomal RNAs (rRNAs) take place in the nucleolus together with assembly of pre-ribosomal particles that are subsequently exported to the cytoplasm to form mature ribosomes (7). The nucleolus's ribosome biogenesis function makes it an important stress-sensor as perturbation of this pathway, commonly referred to as nucleolar stress, can activate p53-dependent and -independent mechanisms to induce cell cycle arrest or even cell death (9–11). Defects in ribosome biogenesis affect the ageing process and manifest in a group of human disorders commonly called ribosomopathies (7,12,13). In addition, the nucleolus influences overall nuclear architecture and can regulate transcriptional activity in other regions of the genome through their localization to perinucleolar heterochromatin (14–16). Furthermore, the nucleolus retains a large number of proteins and therefore influences their abundance in other cellular compartments. Sequestration or release of proteins impacts a range of cellular processes including DNA repair and telomere maintenance (17–19). This wide range of biological processes, essential for cell survival and human health, emphasizes the need to preserve nucleolar function and ribosomal DNA integrity.

### rDNA

**Figure 1.**
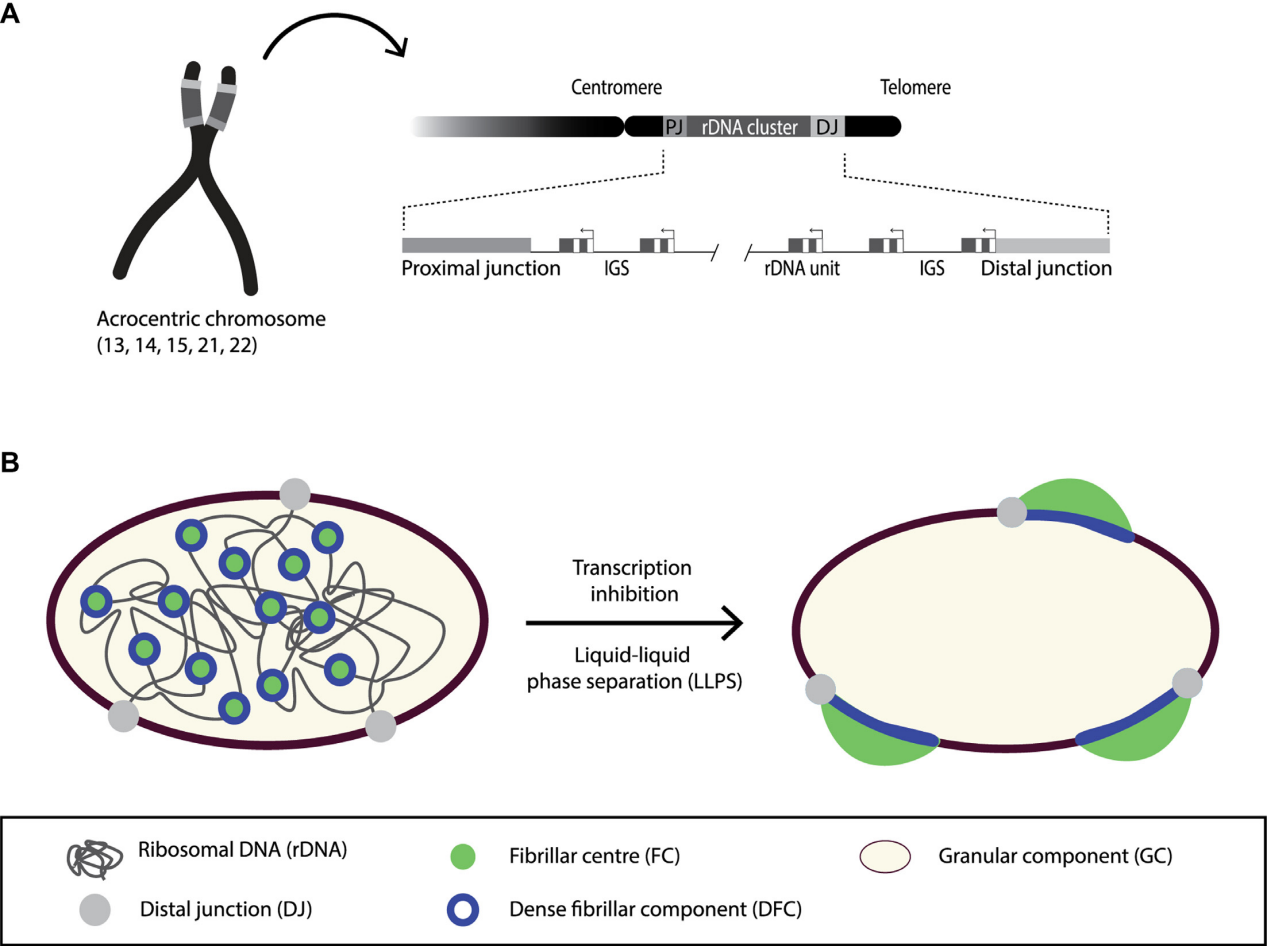
rDNA organization. (**A**) In human cells rDNA clusters are found on the short arm of the acrocentric chromosomes (chromosome 13, 14, 15, 21 and 22). rDNA clusters are flanked by the proximal junction (PJ) on the centromeric side and the distal junction (DJ) on the telomeric side. Multiple ribosomal RNA genes are found in each rDNA cluster separated by the intergenic spacer (IGS) sequences. (**B**) Left panel: an illustration of a transcriptionally active nucleolus with fibrillar centres (FC), dense fibrillar components (DFC) and the granular component (GC) associated with progressive stages of ribosome biogenesis. The DJ is located at the nucleolar periphery with rDNA looping into the nucleolus. Nucleolar sub-structures are dependent on ongoing RNA Pol I transcription and represent immiscible liquid phases. The illustration is inspired from nucleolar electron microscopy images but rDNA may not be accurately depicted as its spatial organization is yet unclear. Right panel: inhibition of RNA Pol I transcription triggers restructuring of the rDNA and rDNA-associated proteins in the nucleolus through liquid-liquid phase separation. rDNA then becomes condensed in nucleolar caps at the nucleolar periphery.

Human cells contain hundreds of ribosomal RNA genes (rDNA) dispersed in clusters on the short arms of the acrocentric chromosomes (chromosomes 13, 14, 15, 21 and 22). The nucleolus originates from active nucleolar organizer regions (NORs) that are clusters of ribosomal RNA genes (20–22). One nucleolus can contain rDNA from clusters placed on different chromosomes. rDNA clusters are flanked by specific sequences: the proximal junction on the centromeric side, and the distal junction on the telomeric side. The distal junction is important for nucleolar organization and has been proposed to function as an anchor at the nucleolar periphery (Figure 1A) (23). The ribosomal RNA gene encodes the 47S pre-rRNA transcript that is processed and modified to form the 18S, 5.8S and 28S rRNAs incorporated into ribosomes. A 30 kb long intergenic spacer (IGS) containing regulatory elements is found in between rDNA units (23). Active rDNA is highly transcribed by the RNA Polymerase I (RNA Pol I), and rRNA accounts for up to 60% of the cellular transcription in eukaryotic cells (24,25). A chromatin composition specific to the nucleolus may assist and regulate these very high levels of transcription (26,27).

### Nucleolar structure

Structurally, the nucleolus can be divided into three sub-compartments: the fibrillar centre (FC), the dense fibrillar component (DFC) and the granular component (GC) (28) (Figure 1B). The organization of the nucleolus is tightly linked to ribosome biogenesis: rRNA transcription occurs at the transition between the FC and the DFC, whereas processing of pre-rRNAs and ribosome assembly progressively takes place through the DFC and the GC region (7,29,30). Nucleoli exhibit a liquid-droplet behaviour in which the different nucleolar compartments represent immiscible liquid phases (31,32) and this self-organizing structure is driven by rRNA transcription (33–35). Inhibition of RNA Pol I triggers a large-scale structural reorganization of the nucleoli. This so-called nucleolar segregation is defined by the translocation of rDNA repeats and associated proteins from the nucleolar interior to the periphery, where they form focal structures called nucleolar caps, with each cap generally representing a single NOR (Figure 1B) (23,36–38). Inhibition of RNA Pol I transcription allows multiple FC/DFC foci to merge and move to the surface of nucleoli forming nucleolar caps with DFC dividing the incompatible FC and GC (36). A bi-phasic structure is maintained in nucleolar caps, where the DFC is facing the GC (remaining in the nucleolus) and the FC is projected into the nucleoplasm (36).

### rDNA: an intrinsically unstable genomic region

The unique nature of the rDNA makes it prone to various types of DNA damage. The high rRNA transcription levels are likely to generate transcription-replication conflicts known to cause DNA damage and chromosomal rearrangements (39,40). The repetitive nature of rDNA also makes it vulnerable to faulty recombination, leading to lengthening or shortening of arrays and eventually to copy-number instability (41). The existence of molecular mechanisms that regulate expansion and contraction of the rDNA has been shown in yeast (42,43) but if such pathways are also found in mammalian cells is yet unclear. Furthermore, the close proximity of multiple rDNA-containing chromosomes in one nucleolus may put the clusters at risk of inter-chromosomal recombination that can cause genomic instability (44,45). The high GC-content of rDNA can also increase G-quadruplex formation, contributing to replication stress and potentially double-stranded breaks (DSBs), at least in a mutational background (46,47).

### A mutational hotspot in cancer

Genome instability is a hallmark of cancer, and the rDNA is one of the most frequently rearranged chromosomal regions in solid tumours (48,49). rDNA rearrangements, including insertions, translocations and amplifications, are for example common in Hodgkin's lymphoma (50).

Several cancer-associated mechanisms challenge rDNA and cause DNA lesions, potentially explaining the high frequency of rearrangements and compromising overall genome integrity. Oncogene activation and loss of tumour suppressors can cause replication stress that is particularly dangerous for so-called fragile sites in the genome as they break more frequently under such conditions. rDNA contains early replicating fragile sites and is therefore challenged by such genetic changes (51). Oncogene activation and the loss of tumour suppressors also frequently upregulate rRNA transcription (52–54) and may contribute to rDNA instability either by R-loop accumulation, interference between transcription and replication or repair, or potentially through loss of protective silent rDNA (55–57). Defects in DNA repair pathways are another cancer-associated feature that impacts rDNA stability. Patients with Bloom Syndrome and Ataxia telangiectasia, caused by mutations in the repair proteins BLM and Ataxia telangiectasia mutated (ATM) respectively, have strong predisposition to cancer and rDNA instability (58). Furthermore, many cancers exhibit pronounced rDNA array instability, often with a reduced rDNA copy-number compared to normal tissue (59–62). However, the extent to which rDNA instability is a driver of cancer development is still unclear.

### The DNA damage response

To understand and discuss how the nucleolar response to DNA damage has specialized to meet the needs of this particular cellular compartment, we will summarise the mechanisms that cells employ elsewhere in the genome to minimize the consequences of DSBs. DSBs can arise as a consequence of replication stress or be induced by ionizing radiation (IR), chemicals or enzymes (63–65). To safeguard the genome against DSBs, mammalian cells have evolved a complex network of pathways collectively called the DNA damage response (DDR). This network includes break detection mechanisms, DNA repair pathways and cell cycle checkpoints that become activated in response to a DSB. The initial step of the DDR is the recognition of the break and the activation of ATM and Ataxia telangiectasia and Rad3-related (ATR) kinases (66). Following detection, these kinases phosphorylate a broad range of targets and initiate signalling cascades that regulate various cellular processes including DNA replication, transcription, cell cycle progression and senescence or apoptosis if the damage is beyond repair (67,68).

Repair of DSBs is mainly conducted by one of two pathways; non-homologous end joining (NHEJ) or homologous recombination (HR). NHEJ involves a fast but potentially mutagenic resealing of the DNA initiated by the binding of Ku70/Ku80 to the DSB-ends (69). Ku70/Ku80 promotes binding of several other NHEJ factors including DNA-dependent protein kinase catalytic subunit (DNA-PKcs), DNA Ligase 4 and X-ray repair cross-complementing protein 4 (XRCC4) facilitating rapid sequence-independent repair of the break (69).

Alternatively, the other major repair pathway, HR, can be activated. HR is slower and requires a homologous DNA sequence to act as a template for accurate repair (70). Although NHEJ is less accurate than HR and can lead to loss of genetic information, it can occur throughout the cell cycle, whereas HR is generally restricted to S and G2 phases, when a sister chromatid is available (65,69,71,72). One of the first steps towards HR is initiation of resection by the MRN-complex, consisting of MRE11, RAD50 and NBS1 and CtIP (73). Resection initiation is regulated by cyclin-dependent kinases (CDKs) throughout the cell cycle (74). CtIP activation in S-phase is mediated by CDK phosphorylation, and leads to DNA resection, single stranded DNA (ssDNA) generation (promptly coated by RPA), and breast cancer type 1 susceptibility protein (BRCA1) recruitment in response to DSB formation (75). DNA end-resection is an important event in the repair pathway choice and commits cells to HR while inhibiting NHEJ (74). For a comprehensive review see Hustedt *et al.* (74).

The MRN-complex also functions as a scaffold for ATM activation. A direct interaction between the C-terminus of NBS1 and ATM tethers ATM to the break site (76). ATM phosphorylates many targets, amongst them the histone variant H2AX, referred to as γH2AX after phosphorylation (77). γH2AX provides a docking site for various factors, including the mediator of DNA damage checkpoint protein 1 (MDC1) (77). At the site of damage MDC1 interacts with the MRN complex (78), ensuring increased MRN accumulation at the break site and thereby creating a positive feedback loop amplifying ATM recruitment (77). Multiple modifications of the surrounding chromatin lead to the formation of ionizing radiation induced foci that facilitate accumulation of proteins involved in processing and repair of the DSB (65).

### DDR in nuclear space

In the last decade, it has become clear that the response to DSBs is not uniform across the genome. Technical advances have allowed investigation of specialised DDR pathways associated with specific chromatin states or nuclear compartments (79–82): in particular, our understanding of how DSBs are detected, processed and repaired in the nucleolus has advanced significantly. In the sections below these findings will be discussed to give a comprehensive understanding of the nucleolar responses to DSBs and how they protect the integrity of rDNA.

## THE NUCLEOLAR-DNA DAMAGE RESPONSE

The nucleolar-DNA damage response (n-DDR) can induce rapid inhibition of RNA Pol I transcription, formation of nucleolar foci, nucleolar restructuring and formation of nucleolar caps. Below we review each step, including the most recent findings. We also propose a model of how inhibition of nucleolar transcription contributes to nucleolar cap formation under different conditions and highlight outstanding questions, related to the coordination of nucleolar cap formation, repair and cell cycle stage, that can progress our understanding even further.

### Regulation of nucleolar transcription in response to rDNA DSBs

DSBs in nuclear chromatin induce ATM-dependent inhibition of RNA Pol I *in trans*, causing a global and transient transcriptional inhibition of all nucleoli within the cell (83). In mice cells it was demonstrated that when DSBs occur in rDNA, the n-DDR is able to act locally, with an ATM-dependent *in cis* mechanism only decreasing transcription of the affected nucleolus (84). Recent data using human cells demonstrated that in some cases the inhibition is restricted even further, repressing transcription in only part of a nucleolus (85–87). These findings raise the questions of what mechanisms control the spreading of the inhibitory signal in the nucleolus and whether it can be restricted to single rDNA units, single NORs or if chromatin topology influences its range of action (88). Future studies taking advantage of high-resolution technologies can provide additional insights into these questions.

**Figure 2.**
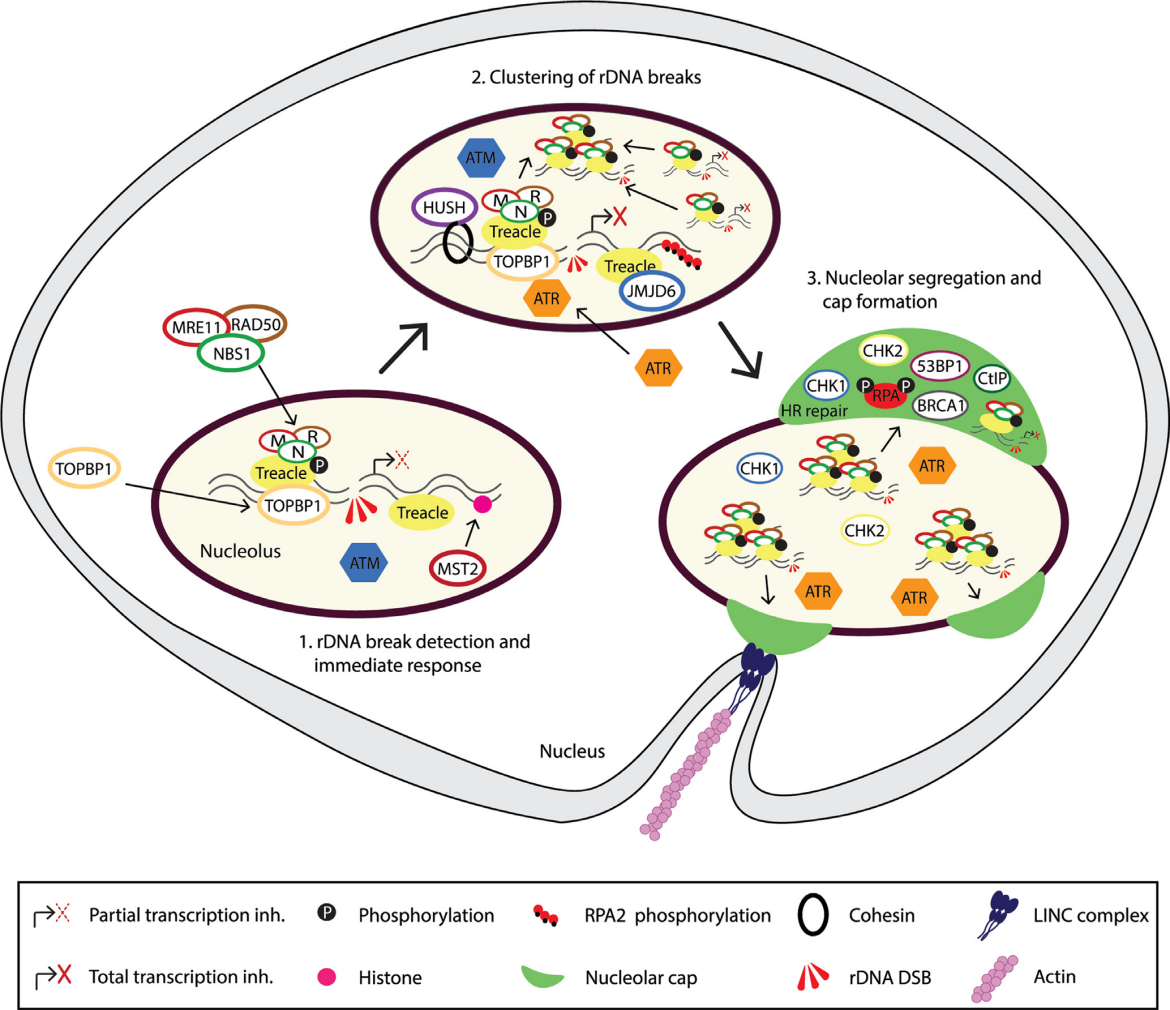
The response to rDNA DSBs. Upon DSBs in the rDNA, ATM becomes activated and initiates the inhibition of nucleolar transcription. A central ATM target is the nucleolar protein Treacle that recruits the MRN-complex and TOPBP1 to activate the ATR kinase. ATR augments the inhibition of nucleolar transcription and promotes nucleolar cap formation. Additional factors, such as the cohesin/HUSH complex, RPA2 phospho-serine 33, JMJD6 and the LINC/actin pathway are also required for nucleolar cap formation under certain conditions. Furthermore, downstream kinases like MST2 and CHK1/CHK2 can contribute to transcriptional inhibition. In nucleolar caps rDNA DSBs are recognized by HR-associated repair factors including CtIP, BRCA1 and RPA2 phospho-serine 4/8.

When ATM becomes activated in the nucleolus it targets a number of nucleolar proteins (89–91). The nucleolar protein Treacle (also referred to as TCOF1) (92) has emerged as a particularly important regulator of transcriptional activity after DSBs. Upon nucleolar DSBs, Treacle recruits the DNA damage protein NBS1 and both are required for nucleolar transcriptional inhibition (Figure 2) (83,84,93). The recruitment of NBS1 to nucleoli requires combined phosphorylation of SQ/TQ sites by ATM and phosphorylated SDT-like motifs on the N-terminus of Treacle to bind the FHA/BRCT domains of NBS1 (83,93), leading to accumulation of NBS1 in nucleolar foci (83,85,93,94).

ATM, however, was recently demonstrated to be insufficient to induce a complete transcriptional shut-down with ATR activity also being required for a complete response (85,93). The role of ATR in regulation of nucleolar transcription has also been reported in other contexts than DSBs (95,96). Interestingly, the activation of ATR after rDNA DSBs is also dependent on Treacle, but in this case through a Treacle-dependent recruitment of DNA Topoisomerase II Binding Protein 1 (TOPBP1) to the nucleolus (93). TOPBP1 binds to the ATM-phosphorylated C-terminal domain of Treacle, with Treacle simultaneously binding NBS1, and NBS1 being required for TOPBP1 recruitment (Figure 2).

In response to rDNA DSBs, NBS1 is recruited to rDNA as part of the MRN-complex, known to process DSB-ends and to contribute to ATR activation (85,93). Depletion of the MRN-subunit MRE11 or inhibition of ATR results in very similar phenotypes including defects in inhibition of rRNA transcription, abrogation of rDNA translocation and accumulation of rDNA foci inside the nucleolus (85,86). As the MRN-complex is acting upstream of ATR activation (85), a resection-mediated role of the MRN-complex in ATR activation was therefore investigated (93). Surprisingly, it was found that ATR activity is required for the formation of nucleolar RPA foci, a commonly used marker for resection and ssDNA (93). In further support of this finding, depletion of the end-resection factor CtIP does not abrogate nucleolar segregation but only influences rDNA repair within nucleolar caps (85,93). These findings suggest that resection is not required for ATR activation and thus the mechanism underlying ATR activation in the nucleolus requires further investigation.

Recent studies indicate that ATM and ATR are not the only kinases activated upon DSBs in the nucleolus. Inhibition of the Checkpoint kinases 1 and 2 (CHK1/CHK2) by small-molecule inhibitors was recently shown to partially compromise transcriptional inhibition (93). The effector kinases CHK1/CHK2 may therefore contribute to transcriptional silencing downstream of ATM/ATR.

The MST2 kinase was also shown to induce transcriptional inhibition via ATM-dependent post-translational modifications of histones. MST2 phosphorylates histone H2B on serine 14 after rDNA DSBs. This response was rapidly and transiently activated in the nucleolus and required for rRNA transcriptional inhibition after rDNA DSB (27).

In addition, ATM may also play a role in transcriptional inhibition through a process involving the cohesin subunits SMC1 and SMC3 (97). Cohesin interacts with the MRN complex and ATM-mediated phosphorylation of cohesin is dependent on NBS1 (97,98). A recent study showed that transcriptional inhibition of the rDNA is regulated through the cohesin complex, which recruits the Human Silencing Hub (HUSH) complex and Suv39H1/2 methyltransferase to introduce H3K9me3 repressive chromatin mark (86), thereby allowing transcriptional shutdown. Depletion of subunits from these complexes impairs rDNA transcriptional shutdown and subsequent nucleolar cap formation upon nucleolar DSB induction (86).

These recent studies have revealed the molecular complexity underlying the inhibition of rRNA transcription triggered by rDNA DSBs (Figure 2). Numerous confirmed targets of ATM/ATR are however present in the nucleolus (89,90) and further investigations are therefore needed for an even more comprehensive understanding of these processes. Future studies will also shed light on the importance of transcriptional inhibition for genome stability. Lack of transcriptional inhibition outside the nucleolus has been associated with large-scale chromosomal rearrangements and translocations and further studies will determine if failure to repress transcription in the nucleolus can have similar consequences, especially as rDNA is found on several chromosomes (99).

### Uncoupling transcriptional shut-down and cap formation

rDNA segregation and nucleolar cap formation has previously been suggested to be a passive process occurring as a consequence of transcriptional arrest (79), as nucleoli form through transcription-mediated liquid-liquid phase separation (31,32,35). This view is in agreement with studies using the endonucleases I-PpoI and Cas9 to induce DSBs in rDNA that reported consistent cap formation after DSB induction in the majority of cells (79,85,93). Recent evidence, however, has shown that under conditions where DSBs were induced by the AsiSI endonuclease, DSB-induced cap formation was restricted to S and G2 phases while transcriptional inhibition occurred during all stages of the cell cycle (86). In this study, the Linker of Nucleoskeleton and Cytoskeleton (LINC) complex was found to mediate nucleolar reorganization through nuclear envelope invaginations that require the actin network (see Figure 2) (86). Interestingly, rDNA stability was also shown to depend on association with the nuclear envelope in yeast, suggesting that this mechanism may be conserved (100). It should be noted however that the AsiSI enzyme induces a large number of DSBs outside the nucleolus (101), and further investigations using rDNA-targeted DSBs should be conducted to verify that the reported mechanism is not influenced by the canonical DDR or by competition between the two DDR-branches. In agreement with the study above, another study reported that upon depletion or knock-out of the histone demethylase JMJD6, an interaction partner of Treacle, transcriptional repression occurred but cap formation was impaired after rDNA damage (102). DSB mobilization was however consistently associated with transcriptional inhibition, suggesting that it is a prerequisite for segregation and cap formation, even if other factors may also be needed.

**Figure 3.**
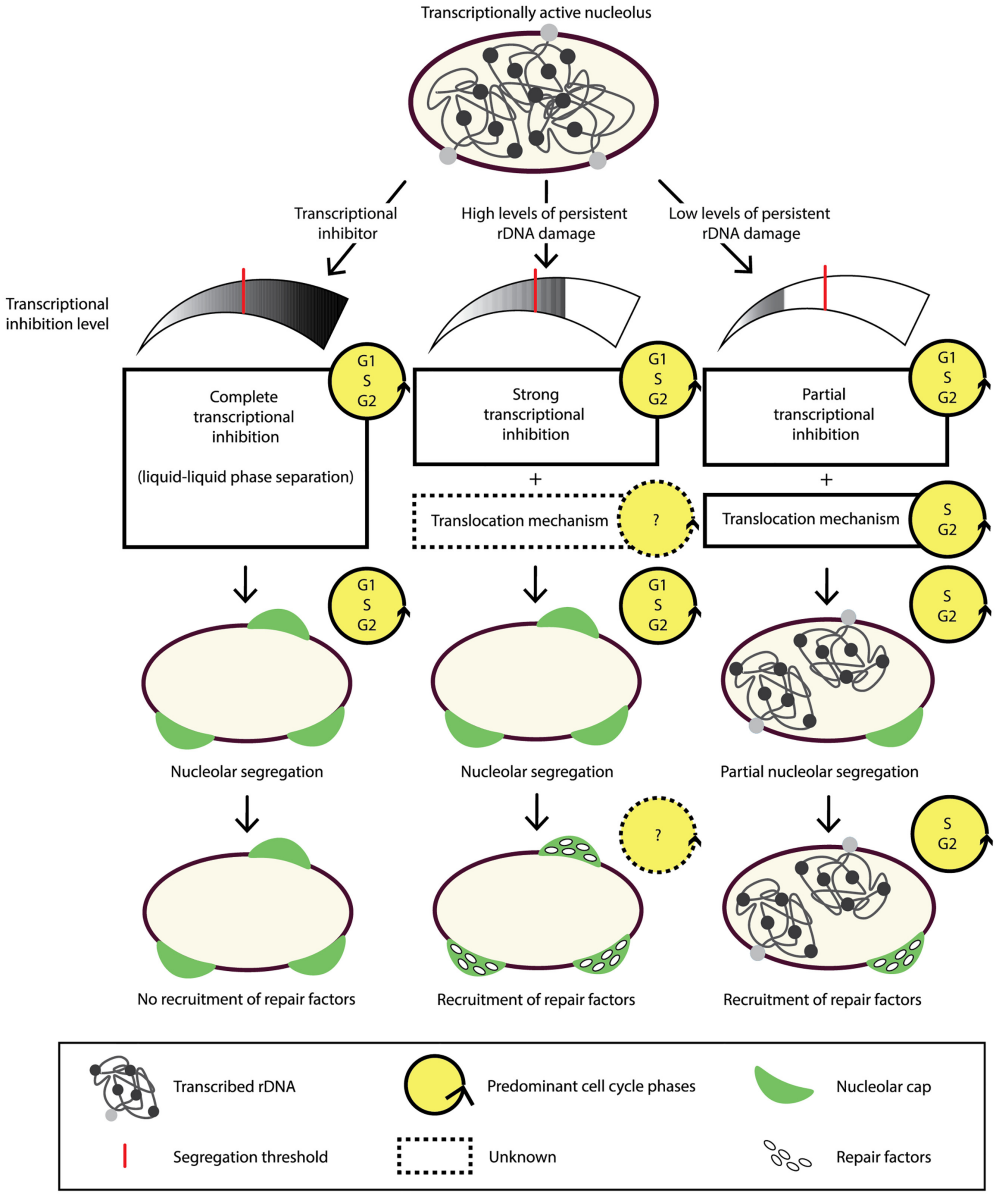
Proposed model for nucleolar segregation and cap formation. Left panel: transcriptional inhibitors can shut down nucleolar transcription and induce LLPS-mediated cap formation in all cell cycle phases without recruitment of repair factors. Middle panel: persistent DNA damage signalling inhibits nucleolar transcription to an extent that exceeds a threshold where nucleolar segregation is induced irrespective of the cell cycle phase. Under such conditions, other cellular mechanisms may assist rDNA segregation and cap formation. Furthermore, if cell cycle phase influences recruitment of repair proteins to nucleolar caps is as yet unclear. Right panel: low level of DNA damage signalling does not trigger immediate nucleolar segregation but depends on other cellular pathways for rDNA translocation and nucleolar cap formation. These caps are primarily formed in S/G2 phases where HR-associated repair factors are available.

In summary, these findings have questioned the previously dominant idea of nucleolar segregation as a passive process occurring solely as a consequence of transcriptional inhibition. New data have revealed an additional layer of complexity where, under certain conditions, nucleolar DSBs may only translocate to the nucleolar periphery as cells enter S or G2 phases. This concept is in agreement with previous data found in yeast (103). The underlying differences between these responses are as yet unclear but the load of damage and persistence of the breaks likely influence nucleolar segregation (for a recent review see Vitor *et al.*, 2020 (65)). We therefore propose a model where persistent DNA breaks result in a signalling response that exceeds a threshold and inhibits transcription in the entire nucleolus (Figure 3) in agreement with data presented previously by the Greenberg lab (104). Transcriptional inhibition then triggers cell cycle-independent nucleolar restructuring and cap formation similarly to that induced by transcriptional inhibitors like ActD (Figure 3) (105). Under conditions with fewer or primarily non-persistent DSBs however, only a local transcriptional inhibition takes place and relocalization of the break depends on complementary mechanisms that are only activated in S/G2 (Figure 3). Future studies using experimental systems where DSB-induction and repair can be monitored in detail will allow a better understanding of the regulation of nucleolar segregation following rDNA DSBs.

### The chromatin context of rDNA DSBs

The n-DDR reflects the different chromatin composition of the rDNA compared to that of nuclear chromatin. One of the major targets of ATM, the histone H2AX, has decreased nucleosome occupancy, particularly in the transcribed region of the rDNA, and limited phosphorylation of H2AX can therefore be detected in the nucleolar interior after ATM activation (41,85,86). The mediator protein MDC1, which is normally recruited to sites of damage by the phosphorylated histone, is excluded from the nucleolar interior (85). Consistent with this observation, NBS1 foci formation upon rDNA damage is independent of H2AX and MDC1 in contrast to the canonical DDR (85). However, Treacle has emerged as a central coordinator of the n-DDR, facilitating MRN recruitment to the nucleolus, binding of TOPBP1 to facilitate ATR activation, interaction with JMJD6 and with a potential link to the cohesin complex via the MRN-complex (41,85,86,102). Treacle is an example of adaption of the n-DDR to a specialized chromatin landscape and a physical environment excluding DDR proteins such as MDC1. Furthermore, these findings place Treacle at the heart of the n-DDR, coordinating multiple branches of the response (Figure 2). The precise role of Treacle in rDNA chromatin remains unclear. Treacle has been described to associate with rDNA through direct binding, and through upstream binding transcription factor (UBF) -dependent and -independent mechanisms (106–108). Further investigations of the structural-functional relationship between rDNA and Treacle will therefore be important to understand the function of Treacle in the n-DDR in greater detail.

### Compartmentalization of rDNA repair

Studies inducing DSBs in rDNA have described a fundamental reorganization of the nucleolus, as discussed above (36,41,85,86,104). As the nucleolus transits from an actively transcribing stage to repressed nucleolar caps, the predominant type of repair likely shifts from NHEJ to HR. Below we discuss the compartmentalization of rDNA repair and the steps required for the individual processes (see Figure 2).

#### NHEJ repair in actively transcribing nucleoli

Immediate repair of rDNA damage is primarily carried out by NHEJ in the nucleolar interior, mitigating the impact on rDNA transcription levels and not causing massive reorganization of the nucleolus (104). This mechanism is dependent on the DNA-PK, XRCC4 and XRCC5 and when abrogated it exacerbates both the transcriptional inhibition response by ATM and the accumulation of downstream repair factors (41,104). Furthermore, it has been demonstrated using I-PpoI that the fraction of broken rDNA loci increased after inhibition of the NHEJ pathway, whereas this was not the case upon inhibition of HR (104). It was therefore suggested that NHEJ repairs the majority of rDNA breaks rapidly in the interior of nucleoli without sustained ATM activation and nucleolar restructuring (104). NHEJ was also found to uphold rDNA integrity in meiosis in *Arabidobsis thaliana* where DSBs occur as part of the meiotic recombination process. rRNA transcription was activated at the onset of meiosis, shielding rDNA from recombination by the HR machinery in the nucleolus, presumably to maintain stable rDNA gene clusters through generations (109). If the nucleolar NHEJ pathway deviates from that operating in nuclear chromatin is currently unclear. More studies focusing on NHEJ in rDNA are required to gain a deeper understanding of how NHEJ functions in a nucleolar context, in particular with regards to its spatial organization (further discussed below).

#### Mobilization of rDNA and nucleolar reorganization

When rDNA breaks persist, they are mobilized to the nucleolar periphery where HR protein assembly can take place. The mobilization of rDNA follows a characteristic pattern: initial formation of small interior n-DDR foci, followed by clustering into larger foci still in the nucleolar interior that eventually translocate to the nucleolar periphery to constitute nucleolar caps (85,86). As mentioned, this translocation of DSBs to the nucleolar periphery is associated with a shift in repair pathway, from NHEJ to HR. At which stage of this process the commitment to HR occurs and what role DNA end-processing plays in rDNA mobilization is emerging from recent data. Data using the AsiSI endonuclease showed accumulation of both total RPA and RPA2 phospho-serine 33 already prior to segregation of rDNA (86). Depletion of RPA2 prevented rDNA segregation and strongly decreased cap formation, demonstrating that RPA is required for nucleolar cap formation (86). This increase in RPA2 phospho-serine 33 is specific for rDNA damage repair and not equally pronounced after transcription inhibition by ActD. Depletion of MRE11 (known to initiate resection) was shown to yield a similar phenotype, with decreased accumulation of RPA2 phospho-serine 33 and reduced cap formation. Depletion of BLM/DNA2 (proteins involved in long-range resection) also reduced phosphorylation of RPA2 but only resulted in a small defect in cap formation. Based on these observations, the authors concluded that resection occurs prior to nucleolar segregation and that it is required for cap formation (86). The requirement of the MRN-complex for ATR activation in the nucleolus should however be taken into account, and that RPA2 serine 33 is an ATR target (110). The depletion of MRE11 could therefore prevent RPA2 phosphorylation and cap formation due to failure to activate the ATR kinase and thereby inhibit nucleolar transcription rather than preventing resection (85,93).

The nucleolar RPA2 phosphorylation pattern in rDNA is also clearly distinct from that elsewhere in the genome. Long-range resection in genomic DNA has been estimated to create >1000 bp overhangs that commit cells to HR (111). In rDNA however, RPA2 phospho-serine 33 occupies the entire 13 kb transcribed region, overlapping with RNA Pol I occupancy (86). Intriguingly, data showed that RPA2 foci formation and translocation of rDNA DSBs to the nucleolar periphery was not affected by depletion of the resection factor CtIP, arguing against resection prior to cap formation (93). Further investigations should determine if RPA-coated DNA is created as a result of resection or if it may have alternative origins. It is not unreasonable to consider whether the very open structure of the transcribed repeats in rDNA could expose stretches of ssDNA that could give rise to the RPA2 phospho-serine 33 upon ATR activation, promoting cap formation.

#### Nucleolar caps: an HR-associated repair compartment at the interface between the nucleolus and the nucleoplasm

When rDNA breaks reach the nucleolar periphery, they are condensed in nucleolar caps, where rDNA DSBs come into contact with HR-associated repair factors including RAD51, RAD52 and BRCA1 (Figure 2) (41,79,85,93,104). The endonuclease CtIP accumulates in nucleolar caps and this event is required for sustained NBS1 and MRE11 accumulation and recruitment of HR factors, such as BRCA1, to nucleolar caps (85). Furthermore, RPA2 phosphorylation on serine 4/8, another marker of resection, accumulates in addition to RPA2 phospho-serine 33 (79,85,93). The accumulation of RPA2 phospho-serine 4/8 is dependent on the nuclease activity of MRE11 and can be abrogated by treatment with the MRE11 inhibitor Mirin (93). The requirement for CtIP, MRE11 nuclease activity and the accumulation of RPA2 phospho-serine 4/8 only in nucleolar caps suggest that the processing of rDNA DSBs in nucleolar caps is qualitatively distinct from the interior RPA2 phospho-serine 33 accumulation. It could also suggest that long-range resection of rDNA occurs at this stage in nucleolar caps (85,86) and not in the interior. In yeast, rDNA resection was shown to be conducted in a hierarchical manner by the three endonucleases encoded by *ast1*, *exo1* and *rad2* (112). Investigation of the involvement of the human homologs in rDNA resection can provide further insight into this process and its regulation in mammalian cells.

Recently, damage-induced small RNAs were identified at the 28S locus of the rDNA(113). These RNA molecules are transcribed by RNA Pol II and possibly regulate resection, RPA coating of single-stranded DNA and loading of HR factors (113–115). To determine how and at what stage of rDNA repair these mechanisms are of importance further studies are required.

Translocation of rDNA breaks to the nucleolar periphery has been proposed to serve as a mechanism to physically separate rDNA repeats from different chromosomes prior to HR to prevent inter-chromosomal recombination (23,38,116). The heterochromatic environment at the nucleolar periphery may also decrease the mobility of the break-ends and thereby limit faulty recombination (117). However, HR repair was reported to lead to loss of rDNA repeats and reduced cellular viability (41) and depletion of HR factors resulted in faster resolution of foci, rescued repeat loss and increased viability, indicating that HR repair of the repetitive rDNA is not without consequences even in nucleolar caps (41).

As mentioned above, the upstream role of NHEJ and the accumulation of HR factors in nucleolar caps suggest a shift in the use of repair pathways as rDNA translocates to the nucleolar periphery. Previous reports failed to detect NHEJ factors in nucleolar caps (79), further supporting this idea. However, NHEJ factors are not trivial to detect by immunofluorescence and additional studies are required to determine if NHEJ contributes to rDNA DSB repair after translocation or if HR is the predominant pathway operating in nucleolar caps.

### Coordination of cell cycle and accumulation of repair factors

Previously, McStay and colleagues proposed a model suggesting that the HR repair pathway is used for rDNA DSBs even in G1, templated *in cis* by repeats from the same NOR (79). The formation of nucleolar caps and accumulation of HR factors including RPA, RAD51, RAD52 and BRCA1 were confirmed by several labs using the CRISPR/Cas9 and the I-PpoI systems, suggesting that the HR pathway can be used for rDNA repair in G1 (41,79,85,93,104). Interestingly, data also using the I-PpoI showed that even though caps formed throughout the cell cycle, RPA2 accumulation in caps was either delayed in G1 versus S/G2 (RPE1 cells) or less pronounced (U2OS cells), suggesting that cell cycle stage does influence the response (93). A cell cycle dependent repair pattern was even more pronounced when examined using the AsiSI endonuclease in U2OS cells to induce DSBs in rDNA. The AsiSI endonuclease induced nucleolar caps in 35% of cells (Compared to 70% in Cas9 and I-PpoI treated cells) and the majority were found in S/G2 phases of the cell cycle. A similar pattern was found for RPA2 phospho-serine 33, suggesting that repair of rDNA DSBs by HR in G1 may not be the bona fide pathway, even if it can be employed (79). Detailed analysis of cell cycle stage and accumulation of individual repair factors will be required to fully understand how this process is regulated (Figure 3) and if HR repair in G1 is distinct from that in S/G2.

### Checkpoint signaling beyond the nucleolus

DSBs in nuclear chromatin efficiently activate cellular checkpoints, but recent data suggest that the n-DDR may also deviate in this regard. rDNA damage generated by CRISPR-Cas9 in U2OS and HEK 293T cells did not induce global cellular phosphorylation of the DDR effector proteins CHK1, CHK2 and KAP1, or activate the G2/M checkpoint (85). However, as previously mentioned, the CHK1/CHK2 kinases were activated after DSB-induction using the I-PpoI endonuclease, and chemical inhibition of CHK1/CHK2 compromised nucleolar transcriptional inhibition (93). These data suggest that the CHK1/CHK2 kinases are activated as part of the n-DDR in the nucleolus but that the signal is potentially contained within individual nucleoli, as has also been demonstrated for the ATM and ATR kinases (79,84). In contrast, a study examining rDNA damage in RPE1 cells noticed a strong inhibition of mitotic entry after DSB. Further investigations should therefore clarify the underlying differences and if variation between cell lines occur (41).

## THE NUCLEOLAR DDR AND GENOME INSTABILITY

### The role of the n-DDR in genome stability and cell viability

The factors involved in the n-DDR have proven to be essential to maintain genome integrity and thus cellular survival. In this context, recent evidence revealed the existence of phenotypes associated with a dysfunctional n-DDR. Induction of rDNA DSBs in cells depleted of Treacle or MRE11 results in an elevated number of dead cells, increased apoptosis and higher frequency of micronuclei (85,93). Besides Treacle and MRE11, depletion of TOPBP1 was also shown to decrease cell viability upon DSBs generated primarily in the rDNA (93). On the other hand, depletion of the cohesin subunit SMC1 and SMC5 led to decreased rearrangements of rDNA, with a rescue of non-canonical rDNA units and loss of repeats respectively. (41,86). Depletion of BRCA1 also prevented rDNA repeat loss after DSB generation (41). SMC1, SMC5 and BRCA1 all promote HR repair, which suggests that HR repair is potentially harmful as mentioned above and a process that can compromise survival. Further investigations are required to fully understand the impact of defects in n-DDR factors on genome stability and cellular fitness. This will be an important task in order to understand the role of n-DDR factors in pathogenesis, and in particular, in cancer.

### The n-DDR as an anti-cancer target

In the pursuit of new cancer treatments, the potential of the n-DDR as an anti-cancer target should be evaluated. An individual assessment of candidates will likely be required, as depletion of different n-DDR factors has very different outcomes both with regards to cell survival and genome instability.

Several compounds have been developed to selectively inhibit RNA Pol I (118–122). CX-5461 was the first RNA Pol I inhibitor described and also currently the only RNA Pol I inhibitor that has completed a clinical phase I study. From the clinical trial, CX-5461 was found safe and the maximum tolerated dose was determined. CX-5461 prevents the formation of the pre-initiation complex and thereby inhibits RNA Pol I transcription leading to activation of the ribosomal checkpoint and induction of cell death predominantly in tumour cells (52). CX-5461 was also found to be selectively lethal in BRCA1/2 deficient tumours possibly via stabilization of G-quadruplex structures and is currently in clinical phase I for BRCA1/2-deficient tumours (123). A second promising RNA Pol I inhibitor is BMH-21. BMH-21 was identified based on its ability to activate p53 and further studies demonstrated that this was mediated through activation of the ribosomal checkpoint. BMH-21 stalls RNA Pol I transcription and leads to degradation of the RNA Pol I subunit, RPA194, and thereby acts through mechanisms distinct from those of other RNA Pol I inhibitors (124). BMH-21 was demonstrated not to activate the DNA damage response, potentially resulting in less side effects in a clinical context (122). For a detailed and recent review see Ferreira *et al.* (121).

An alternative strategy to consider is the inhibition of early n-DDR factors. When cells are depleted of n-DDR factors such as Treacle and MRE11, they become genomically unstable as described previously. The nucleolar stress that consequently arises can potentially induce cell cycle arrest and even cell death through abrogation of ribosome biogenesis (125,126). This combination of responses makes early n-DDR factors interesting anti-cancer targets for future investigation: more translational and clinical studies are required to test their potential in vivo.

The power of synthetic lethality has been demonstrated clinically and is also relevant in the context of the nucleolus as an anti-cancer target. Many different types of chemotherapeutics target the nucleolus and induce DNA damage or ribosomal stress (125). Oxaliplatin is an example of a common chemotherapeutic that induces ribosomal stress (127) but a large number of other chemotherapeutics have been demonstrated to do so (128). It will be interesting to further investigate if a synthetic lethal relationship exists between such chemotherapeutic compounds and n-DDR factors.

A thorough knowledge of the n-DDR may be used to hamper cancer development and progression and can potentially pave the way to identify new protein targets and treatment strategies for cancer therapy.

### rDNA instability and ageing

In yeast, rDNA instability has emerged as an important aspect of cellular ageing. rDNA instability increases with age, correlates with lifespan and likely contributes directly to the ageing process (42,43,129,130). In human cells the connection between rDNA instability and ageing is less clear. The human premature ageing disease, Werner Syndrome, shows increased frequency of rDNA rearrangements (131) and patients with Lewy body dementia also display alterations of rDNA (132), suggesting that ageing and age-related diseases can be associated with rDNA instability. However, to obtain a more comprehensive understanding of the causative effect of rDNA in the ageing process in humans further studies will be required.

## CONCLUSION

In summary, recent discoveries have significantly extended our understanding of how cells respond to DSBs in rDNA and have placed ATR downstream of ATM as a key regulator of transcriptional inhibition. The nucleolar protein Treacle has emerged as a central coordinator of several branches of the n-DDR with links to both transcriptional inhibition, recovery and DNA repair. To determine how processing of rDNA is conducted, its role in nucleolar segregation and when cells commit to HR, further studies are necessary. To understand nucleolar regulation of HR repair in more depth, additional investigations must be conducted as current data indicate regulation both at the level of cap formation and recruitment of HR-factors to caps (Figure 3). A recent discovery of a potential fourth nucleolar compartment called ‘nucleolus rim’ at the nucleolar periphery may also be of importance to understand regulation of HR in the nucleolus (133). Moreover, these regulatory mechanisms may not apply equally to all types of DSBs for reasons currently unknown. In addition, the previously dominating idea of nucleolar segregation as a passive process has been challenged and nucleolar caps likely require additional cellular pathways to form, in particular under conditions where a DSB occurs in a nucleolus that is still partially transcriptionally active.
